# Breaking down the wall: Solid-state NMR illuminates how fungi build and remodel diverse cell walls

**DOI:** 10.1371/journal.ppat.1013678

**Published:** 2025-11-11

**Authors:** Isha Gautam, Ankur Ankur, Kalpana Singh, Anand Jacob, Tamara L. Doering, Neil A. R Gow, Jean-Paul Latgé, Tuo Wang

**Affiliations:** 1 Department of Chemistry, Michigan State University, East Lansing, Michigan, United States of America; 2 Department of Molecular Microbiology, Washington University School of Medicine, St. Louis, Missouri, United States of America; 3 Medical Research Council Centre for Medical Mycology at the University of Exeter, University of Exeter, Exeter, United Kingdom; 4 Unité des Aspergillus, Institut Pasteur, Paris, France; McGill University, CANADA

## Introduction

The fungal cell wall, an essential organelle that governs growth, has been recognized for centuries as critical to fungal biology, yet its structure and biosynthesis remain poorly understood. Fungal growth and survival depend on the complex dynamics of the cell wall, which is not a static shell but a dynamic, ever-changing structure that support adaptation and interactions with the host and environment [1,2]. Made up of a fascinating mix of polymers of different chemical structures and composition, these walls form intricate networks whose architecture varies widely across species and life stages. Yet, because they are insoluble and complex, peering inside these walls at the molecular level has long been a challenge. To investigate the dynamic complexities of the cell wall, many conventional analytical methods rely on destructive procedures that disrupt its native architecture and therefore cannot provide information about the physical associations between its component parts. To overcome this challenge, we and others are applying solid-state nuclear magnetic resonance (ssNMR) spectroscopy to unlock the secrets of fungal cell walls and reveal their complex organization, the sophisticated interplay of polysaccharides in their native state, and the mechanisms by which they remodel to survive and thrive [3]. This mini-review differs from several recent reviews that have focused on the working principles of ssNMR and its integration with existing biochemical methods for cell wall characterization [3–5]. Instead, we explain how ssNMR has advanced our understanding of fungal cell walls at the molecular level, without the need to physically break them down into component parts. This strategy has revealed both universal design principles and species-specific traits that fungi use to thrive, thus opening exciting new avenues for antifungal research.

## How has ssNMR, as a new tool, advanced our understanding of fungal cell walls?

Understanding the structure of the fungal cell wall in the case of pathogens is critical, since it is a prime target for antifungal drugs, including the echinocandins, ibrexafungerp, GPI-anchor inhibitors such as manogepix, and other inhibitors of polysaccharide synthase and transglycosidase [6–8]. Throughout six decades of research aimed at understanding the fungal cell wall, traditional biochemical methods have relied on harsh treatments, such as alkali extraction [9], which alter the true *in vivo* organization of cell wall polymers. These procedures can be avoided with ssNMR, which directly examine intact—or even living—cells enclosed in a tiny device called a rotor. Each sample typically contains 0.5–100 mg of fungal material, depending on the rotor size (0.7–4.0 mm in diameter) [3,10]. This technique thus can probe native cells in their intact form, by relying on detecting the electronic environment around carbon, nitrogen, and hydrogen nuclei. The key advantage of ssNMR over solution NMR is its ability to investigate non-crystalline and insoluble samples. This technique reveals rich details about the polysaccharides present in a sample, their shapes, motions, associations with water, and how they spatially weave together into a robust polymer network. When combined with conventional chemical analyses of covalent linkages, ssNMR paints a comprehensive picture of how fungal cell walls are assembled and maintained [4].

The application of ssNMR has allowed us to observe not just the static structure of the cell wall, but also how it remodels and adapts over time. By examining changes under growth, stress, or antifungal treatment, we have observed how polysaccharide domains remodel, how hydration affects wall flexibility, and how different fungi adapt their wall composition to survive environmental challenges. Comparative studies across species have also identified both conserved architectural motifs and species-specific adaptations, linking molecular structure to ecological strategy and pathogenicity, deepening our fundamental understanding of fungal biology. Overall, this approach achieves atomic-level resolution of complex biomaterials previously beyond reach, unveiling details that conventional methods could not capture and that are emphasized by the examples discussed in this Pearl.

## Can ssNMR uncover distinct cell wall structures across fungal species and morphotypes?

Fungal cell walls are remarkably diverse across species. While the core components—chitin, glucans, and mannans—are widely conserved, their relative abundance, structural arrangement, and cross-linking patterns vary significantly. High-resolution ssNMR analysis of fungal cell wall structure began in 2018 with the filamentous fungus *Aspergillus fumigatus* [11,12], and has since rapidly expanded to other Ascomycetes, Basidiomycetes, and Zygomycete species. These include: *Aspergillus nidulans*, *Aspergillus sydowii, Aspergillus atacamensis, and Aspergillus destruens* [13,14]; *Cryptococcus neoformans* [15–17]; *Candida albicans* and *Candida auris* [18,19]; *Schizophyllum commune* [20,21]; *Schizosaccharomyces pombe* [22]; *Ganoderma lucidum* [23]; *Neurospora crassa* [24]; and *Mucor* and *Rhizopus* species including *Rhizopus delemar*, *Rhizopus microsporus*, *Rhizopus hiemalis*, *Mucor lusitanicus*, and *Mucor circinelloides* [25]. The result has been a paradigm shift in our understanding of fungal cell wall architecture as highlighted below.

**Fig 1:**
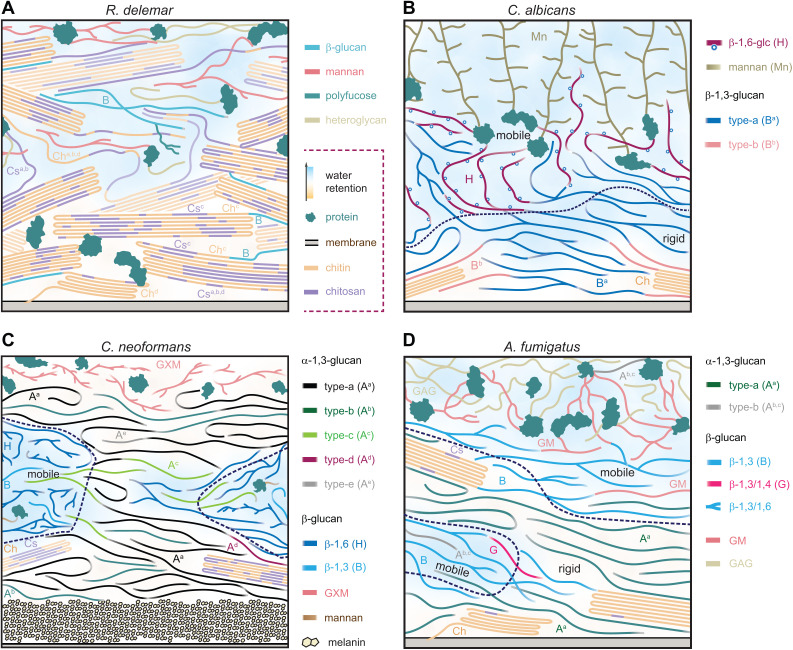
NMR-revised cell wall models in *Rhizopus*, *Candida*, and *Cryptococcus* species. **(A)** Schematic illustration of chitin- and chitosan-rich *R. delemar* mycelial cell walls. Legends in purple dashed lines are applicable to all four panels of this figure. **(B)** Structure of *C. albicans* cell walls, with a mannan-rich outer layer and glucan-/chitin-rich inner domain. Dashed lines separate the rigid core and mobile shell. **(C)** Attachment of capsular carbohydrate GXM and deposition of melanin as mediated by five forms of α-1,3-glucans (types a-e) in *C. neoformans* cell walls. Hydrated mobile domains are highlighted in dashed lines. **(D)** Architecture of 3-day-old *Aspergillus fumigatus* mycelial cell wall, with galactomannan (GM) and galactosaminogalactan (GAG) coexisting with proteins in the outer shell, and chitin, α-glucan, and β-glucan dominating the inner core. Figures adapted from three open-access publications [15,18,25,34].

Excitingly, ssNMR studies have revealed new associations among structural polysaccharides. For example, we recently leveraged our ssNMR expertise to investigate *Rhizopus* and *Mucor* species [25]. These are the main agents of mucormycosis, including COVID-19-associated mucormycosis, which caused severe coinfections during India’s 2021 COVID-19 surge [26]. We found that the cell walls of five *Rhizopus* and *Mucor* species are dominated by crystalline yet highly polymorphic chitin and chitosan (*e.g.,* chitin types a-d, or Ch^a-d^, and chitosan types a-d, or Cs^a-d^, as observed in *R. delemar* and shown in Fig 1A), with each molecule forming four distinct structural sub-types that differ in conformation and hydrogen-bonding patterns—features that can be uniquely resolved by solid-state NMR in intact cells [25]. Unexpectedly, we found that β-glucan, present in limited amounts, binds exclusively to a specific structural form of chitin/chitosan, and that nikkomycin treatment selectively depletes this complex without affecting other three chitin/chitosan forms, leaving overall cell wall integrity largely intact [25]. These findings provide the first in-depth view of carbohydrate structure and nanoscale organization in *Rhizopu*s and *Mucor* cell walls and offer new insights into the structural and biosynthesis complexities of chitin. Importantly, they also demonstrate, for the first time, that selective depletion can target a specific type of chitin among the multiple forms that coexist within the wall, highlighting a functional specificity may be important in future consideration of antifungal strategies.

We found that yeast cells of *C. albicans* and the emerging superbug *C. auris* share similar cell wall architectures. Both species possess an mobile outer layer rich in mannan and a rigid inner layer of tightly associated chitin microfibrils and β-1,3-glucans, supported by a flexible matrix of β-1,6-glucans and additional β-1,3-glucans (Fig 1B) [18,27]. We identified a specific conformational structure of β-1,3-glucans, referred to as the type-b form (B^b^ in Fig 1B), as being responsible for association with chitin microfibrils. This interaction leads to a flattening of the β-1,3-glucan conformation on the microfibril surface, in contrast to the triple-helix structure characteristic of the bulk type-a form (B^a^) [18].

In *Cryptococcus* species, the cell walls are associated with both a polysaccharide capsule and melanin. These, together with the unique cryptococcal cell wall composition, confer innate resistance to cell-wall-targeting antifungals such as echinocandins [28–31]. High-resolution ssNMR analyses revealed the highly polymorphic nature of α-1,3-glucans and their multifaceted roles in these walls: scaffolding melanin and the capsule on opposite faces of the wall, integrating with chitin to provide mechanical strength, and combining with β-glucans to form a flexible, pliable matrix (Fig 1C) [15].

Even within a single species, the fungal cell wall can change dramatically between morphotypes to support shifts in shape, mechanical stability, and host interactions. Recent ssNMR studies on *A. fumigatus* have characterized not only the distinct composition of polysaccharides but also the molecular organization of intact cell walls across developmental stages, including dormant conidia, swollen conidia, germ tubes, and hyphae [11,32]. Dormant conidia have a rigid wall with abundant β-glucan, well-structured chitin, and no galactosaminogalactan. This technique revealed that, as conidia swell, the wall becomes more hydrated, the α- to β-glucan ratio balances, and galactosaminogalactan biosynthesis begins [11]. During germination, chitin embeds in the inner core and separates from glucans, while β-glucans and galactosaminogalactan form a flexible matrix that supports polarized growth and germ tube emergence [11]. Finally, fully developed mycelia feature a mobile outer layer rich in galactosaminogalactan and galactomannan, and a rigid inner layer of β-glucan interlaced with tightly packed chitin and α-1,3-glucans, that forms a hydrophobic core (Fig 1D) [32]. Further, β-1,6-glucan biosynthesis occurs only in the conidia, not in the mycelia, of *A. fumigatus* [33].

## How do fungi remodel their walls under stress or antifungal treatment?

**Fig 2:**
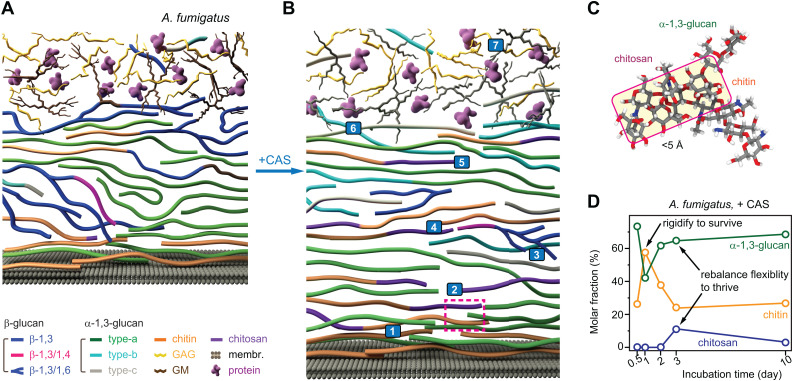
Adaptive remodeling of the *Aspergillus fumigatus* cell wall under caspofungin (CAS) treatment. **(A)** Schematic illustration of cell wall organization in 3-day-old *A. fumigatus* mycelia without treatment. **(B)** Cell wall organization under CAS treatment. Seven major structural changes are highlighted and numbered, and they are discussed in the same sequence in the text. All seven mechanisms are present in the three-day culture, and the numbering does not imply a sequence. The dashed-line region shows the tight packing of molecules, which will be further highlighted in the next figure panel through modeling. **(C)** Tight packing between chitin/chitosan and α-1,3-glucan revealed by microsecond-long all-atom molecular dynamics modeling. **(D)** SsNMR estimation of rigid polysaccharide fractions as a function of culture time with exposure to CAS. Figures adapted from reference [34], an open-access publication.

Integration of recent ssNMR studies with chemical analyses and imaging data has revealed that the *A. fumigatus* mycelial cell wall is organized into two structural domains: a mobile outer layer rich in galactosaminogalactan, galactomannan (GM), and associated proteins, and an inner region consisting of a β-glucan matrix intertwined with dense, hydrophobic domains built from compact chitin and α-1,3-glucans (Fig 2A) [12,32]. When exposed to caspofungin, the fungal cell wall loses much of its water-retaining capacity due to β-glucan depletion, with water accessibility—a property uniquely resolvable by ssNMR—decreasing, while overall thickness increases from ~130 to ~180 nm, likely as a protective adaptation (Fig 2B) [34]. These changes appear to represent universal mechanisms employed by fungi to cope with external stress. For example, halophilic species like *A. sydowii* use similar strategies to survive in hypersaline environments [14], while species such as *C. albicans* exhibit comparable responses when exposed to caspofungin [18].

High-resolution ssNMR further uncovered seven remarkably distinct structural mechanisms, numbered 1–7 in Fig 2B, by which *A. fumigatus* mycelial wall can remodel during three days of caspofungin treatment. These events include the following (numbering does not imply a sequence): (1) the soft β-1,3-glucan matrix disappears under drug exposure, leading to the loss of covalently linked polysaccharide cores such as chitin-β-1,3-glucan-GM and β-1,3-glucan-GM, which are abundant in untreated cells [34]. (2) Chitin biosynthesis is enhanced, reinforcing wall rigidity while preserving its polymorphic structures and associations (Fig 2B). (3) The remaining β-glucans are restructured, showing increased β-1,6-branching and physical association of β-1,3/1,4-glucan linear domains with chitin. (4) The lost interactions between β-1,3-glucan and chitin are now compensated by new stable interactions between α-1,3-glucan and chitin/chitosan, as well as β-1,3/1,4-glucan and chitin/chitosan. These new interactions were observed by ssNMR and confirmed by all-atom molecular dynamics modeling, as evidenced by the stable physical packing within 5 Å in the chemical structure shown in Fig 2C. (5) About one-third of chitin is deacetylated to chitosan, producing partially disordered but well-integrated structures within the compacted chitin-α-glucan complex (Fig 2B). (6) Two new semi-dynamic α-1,3-glucan allomorphs (type-b and type-c) appear, distributed across rigid and mobile phases and adopting altered helical conformations. (7) The cell surface is dramatically remodeled: reduced galactosaminogalactan content lowers the net surface charge, and together with decreased GM branching, weakens the adhesive properties of the cell wall.

Among these changes, those occurring in the rigid core of *A. fumigatus* were found to follow a two-step process. Within 12 hours of exposure to caspofungin, β-1,3-glucan is depleted. This triggers the first remodeling phase: a 2- to 3-fold surge in chitin content, which increases the amount of fibrillar domains and rigidifies the cell wall to withstand stress (Fig 2D) [34]. This was followed by a second phase in which chitin levels return to their original state, compensated by increases in chitosan and α-1,3-glucan; this adjustment provides the flexibility required for the fungus to continue growing and thriving.

We also elucidated the distinct cell-wall-remodeling mechanisms employed by *C. albicans* and *C. auris* in response to echinocandin treatment. *C. albicans* responds with wall thickening and changes in the dynamics of chitin and glucan, whereas *C. auris* maintains wall integrity primarily through upregulation of β-1,6-glucan [18]. Deleting genes homologous to *KRE6*, which is required for β-1,6-glucan synthesis, reduces susceptibility to micafungin and caspofungin, while β-1,6-glucan levels are restored upon echinocandin exposure, thus uncovering the function of this enigmatic carbohydrate in reshaping the cell wall for antifungal response [18].

## What functions do chitin, chitosan, glucans, and mannans serve in wall construction?

Recent ssNMR analyses have also helped us better rationalize the distinct functions of fungal polysaccharides. These functions are derived from the biophysical and structural properties of cell wall polysaccharides—rigidity, dynamics, water retention, and intermolecular interactions—observed across many fungal species. Chitin, the best-understood molecule, forms microfibrils that provide mechanical support and partial crystallinity. It is characterized by high rigidity, as revealed through ssNMR analysis, and serves as the primary structural scaffold of the cell wall [32,35]. Chitosan signals, often found in both rigid and mobile domains, are uniquely abundant under stress; these molecules integrates with chitin to impart partial flexibility to these otherwise rigid domains, a feature crucial for adaptive remodeling and growth [14,25,34]. α-Glucans exhibit a broad distribution in conformational structure, dynamics, and hydration profiles as detected by ssNMR, enabling them to play multiple roles: they act as spacers that stabilize rigid domains by docking on the surface of chitin microfibrils, while also extending into the mobile phase to provide additional flexibility [12,15,32]. This molecule is also broadly distributed across the wall, enabling diverse functions, as seen in *A. fumigatus* and *C. neoformans* [15,32]. β-Glucans, present in diverse linkages, serve both as a covalent cross-linker, as revealed by chemical analyses [9], and as a stabilizer of physical contact with chitin and α-glucan, as revealed by ssNMR data [32]. β-Glucans are typically the best-hydrated molecules within the rigid core of the wall, as shown by ssNMR, allowing them to maintain wall permeability, while their absence can lead to denser, less-permeable domains that act as barriers to small molecules [32,34]. Mannan occupies semi-dynamic positions on the wall surface or in intermediate layers. Under stress, it can rigidify and contact chitin microfibrils, primarily through sidechains, thereby reinforcing the wall while retaining some mobility. This is manifested in ssNMR as a shift of these sidechain signals from the mobile to the rigid phase, where they begin to establish intermolecular correlations with chitin signals [18,32,34]. Notably, the biophysical properties identified by ssNMR for each molecule tend to be largely consistent across different fungal species, suggesting that these molecules, by virtue of their chemical structure, are inherently suited to perform specific functions in cell wall construction. Together, these carbohydrate molecules perform a coordinated, dynamic dance, generating structurally diverse cell walls across species, morphotypes, and environmental conditions.

## How can ssNMR support the understanding of fungal pathophysiology, virulence, and the development of antifungal strategies?

Looking ahead, ssNMR has great potential to advance our understanding of fungal pathogens, especially in disease-relevant contexts. For example, it can enable in situ evaluation of immune effectors, such as reactive oxygen species or antimicrobial peptides [36], by directly monitoring molecular-level changes in fungal cell wall architecture. This technique will allow monitoring of global cell wall modifications during phagocytosis and broaden our understanding of how the cell wall changes in response to antifungal drugs or during the life cycle. The continued application of ssNMR to diverse fungal species, including clinical isolates with established antifungal resistance and tolerance and emerging pathogenic fungal species, will provide valuable insights into both conserved and species-specific responses to treatment.

The applications of ssNMR is also being extended to studying fungi within complex microbial communities. This technique can be used to reveal how fungal cell walls respond to bacteria and other microbes in the microbiome [37]. Examining how environmental factors such as stress, nutrient availability, or host interactions influence cell wall structure will further be essential for understanding fungi in their natural contexts.

Another important consideration is that most ssNMR datasets collected to date for analysis of glycans also capture signals from proteins and lipids [11,14,32]. These signals have not yet been analyzed in detail, partly because they may originate from multiple intra- and extracellular sources. With the development of reliable calibration methods or appropriate controls, these rich datasets could one day be used to probe the roles of structural proteins, enzymes, and membranes in fungal biology and pathogenesis.

The observed structural polymorphism of polysaccharides, in which chemically identical molecules display a wide range of ssNMR peaks reflecting variations in conformation and hydrogen bonding, may be linked to the complexity of their biosynthesis [35]. The production of a single type of carbohydrate often involves multiple enzymes, such as the many chitin synthases, that coordinate with numerous other proteins during biosynthesis. This spectroscopic technique may help decipher the functions of individual enzymes that contribute to such processes, by examining structural outcomes in corresponding mutants, including changes in carbohydrate composition and polymorphic forms [18,32,33].

Despite the power of ssNMR, it has several limitations, which we recently summarized in a technical review [38]. Here, we would like to highlight several major bottlenecks. First, the technique is limited by its low sensitivity and typically requires weeks to months of NMR instrumental time for each project. Second, data analysis is highly time-consuming and demands substantial expertise. This consideration is especially critical for species that have not yet been studied using ssNMR, as resonance assignments must be rigorously validated, often through comparative analyses of mutants and supporting chemical assays. Third, as ssNMR is more of a biophysical method than a bioanalytical technique, and the experiments must be carefully selected or even tailored to the specific objectives of each project. Therefore, at present, ssNMR studies are feasible mainly for topics of the highest urgency and typically demand the involvement of experienced biomolecular ssNMR experts. We expect these limitations to be mitigated in the future by the systematic documentation of the ssNMR fingerprints of biomolecules across key fungal species, the continued development of sensitivity-enhancing methods that accelerate data acquisition, and the availability of semi-automated approaches that streamline data analysis.

## Conclusions

Building on decades of insights from genetics and classical chemistry, the advent of ssNMR now allows us to enter a new era of fungal cell wall research, offering unprecedented molecular-level resolution of its dynamic architecture. This approach complements prior work by revealing structural details and interactions that were previously inaccessible, and it can be further integrated with other advanced techniques, such as CryoEM, to provide a comprehensive view of cell wall organization [39,40]. Together, these advances will connect molecular structure to biological, ecological, and clinical outcomes, opening transformative avenues in antifungal research and fungal biology and illuminating the mechanisms that underly the remarkable adaptability and survival strategies of fungi.
